# Identification of Endogenous Controls for Analyzing Serum Exosomal miRNA in Patients with Hepatitis B or Hepatocellular Carcinoma

**DOI:** 10.1155/2015/893594

**Published:** 2015-02-26

**Authors:** Yi Li, Liqun Zhang, Fei Liu, Guiming Xiang, Dongneng Jiang, Xiaoyun Pu

**Affiliations:** Department of Clinical Laboratory, Xinqiao Hospital, Third Military Medical University, Chongqing 400037, China

## Abstract

Serum exosomal microRNAs (miRNAs) have received considerable attention as potential biomarkers for diagnosing cancer. The canonical technique for measuring miRNA transcript levels is reverse transcription quantitative polymerase chain reaction (RT-qPCR). One prerequisite for validating RT-qPCR data is proper normalization with respect to stably expressed endogenous reference genes. However, genes that meet all of the criteria of a control gene for exosomal miRNAs have not yet been identified. To find out the control gene for exosomal miRNAs, we evaluated the expression stability of 11 well-known reference genes in circulating exosomes. In this study, we found that the combination of *miR-221*, *miR-191*, *let-7a*, *miR-181a*, and *miR-26a* can be an optimal gene reference set for normalizing the expression of liver-specific miRNAs. This combination enhanced the robustness of the relative quantification analyses. These findings highlight the importance of validating reference genes before quantifying target miRNAs. Furthermore, our findings will improve studies that monitor hepatitis progression and will aid in the discovery of noninvasive biomarkers to diagnose early stage HCC.

## 1. Introduction

Exosomes are 40–100 nm diameter membrane-bound microvesicles of endocytic origin that are released from different cell types under both normal and pathological conditions. Exosomes have been identified in body fluids such as urine, amniotic fluid, malignant ascites, saliva, and blood [1, 2]. Exosomes have pleiotropic biological functions, including roles in the immune response, antigen presentation, intracellular communication, and the transfer of RNA and miRNA [2, 3]. The tetraspanin protein family members CD63 and CD9 are frequently located on the surface of exosomes [4]. Therefore, tetraspanin proteins have been used as markers to identify exosomes.

It has been reported that the majority of serum miRNAs are enriched in exosomes. Exosomal miRNAs could serve as valuable noninvasive biomarkers for distinguishing the type and grade of liver inflammation [5]. Additionally, exosomal miRNAs have high potential utility in the clinical diagnosis of various cancers. Human tumor-derived epithelial cell adhesion molecule- (EpCAM-) positive exosomes circulating in blood have been detected by specialized miRNA expression profiling as promising biomarkers of ovarian cancer [6] and lung cancer [7]. Wang and colleagues determine that exosomal* miR-21* expression is useful for diagnosing HCC [8].

Currently, stem-loop reverse transcription quantitative real-time polymerase chain reaction (RT-qPCR) is widely used to quantitatively analyze circulating miRNAs [9]. More importantly, proper normalization of RT-qPCR data based on stable reference genes is critical for miRNA quantitation, because variations can stem from many sources, such as differences in sample procurement, stabilization, RNA extraction, and target quantification. The above-described differences are not a consequence of the disease state itself [10]. Therefore, identification of optimal genes that are stably expressed, irrespective of treatment, is necessary to define reference genes for normalizing exosomal miRNA expression.

Previous studies have demonstrated that none of the commonly used reference genes for normalization are universal for all tissue types or experimental situations [11–13]. Suitable reference genes have typically been identified for different native tissues and body fluids. In the present study, we aimed to identify potential reference genes suitable for the transcript normalization of serum exosome miRNAs in patients with CHB and/or HCC. These reference genes will enable more accurate and reliable RT-qPCR normalization for hepatopathy exosomal miRNA expression studies.

## 2. Materials and Methods

### 2.1. Ethics Statement

The Medicine Ethics Committee of Second Affiliated Hospital of the Third Military Medical University approved all aspects of this study in accordance with the Helsinki Declaration. All the patients or their guardians provided written informed consent.

### 2.2. Serum Preparation

Preoperative blood from 50 HCC patients was collected at the Second Affiliated Hospital of the Third Military Medical University (Chongqing, China). Blood was collected from 50 CHB patients without HCC and 50 healthy subjects prior to antiviral treatment at the Second Affiliated Hospital of the Third Military Medical University (Chongqing, China). A biopsy was performed to diagnose HCC. Chronic HBV infection was defined as the persistence of HBV surface antigen in the bloodstream for at least 6 months. Healthy controls who were not infected with HBV, HCV, or HIV, had normal liver function tests, and had no history of liver disease were selected. The clinical characteristics of the study participants are presented in Table 1.

All samples were taken from January to August 2014. The peripheral blood samples were collected in 5 mL Vacutainer SST Plus Blood Collection Tubes (Becton, Dickinson and Company, USA). Samples were incubated at room temperature for between 30 min and 2 h. The tubes were centrifuged at 1,500 g for 10 min, and the serum samples were aliquoted and centrifuged again at 2,000 g to completely remove any remaining cells. The serum samples were stored at −80°C until further processing for exosome isolation.

### 2.3. Exosome Preparation

Two hundred and fifty microliters of serum was mixed with 66 *µ*L of ExoQuick exosome precipitation solution. Exosome isolation was performed according to the manufacturer's protocol (SBI System Biosciences, USA). Briefly, the samples were incubated at 4°C for 30 min and then centrifuged at 13,000 rpm for 2 min. The protein-rich supernatant was removed, and the exosome-rich pellet was retained for RNA extraction or Western blot.

### 2.4. Transmission Electron Microscopy

Electron microscopy was performed on serum exosome samples at the Biomedical Analysis Center, Third Military Medical University, Chongqing, China. Samples were prepared as described by Thery et al. [14]. Briefly, the exosomal fraction was mixed 1 : 1 with 4% paraformaldehyde in phosphate-buffered saline (PBS), and then the samples were transferred onto Formvar/carbon-coated copper grids and dried at room temperature for 20 min. After a quick wash, the grids were fixed with 1% w/v glutaraldehyde in PBS and washed several times in distilled water. The samples were contrasted with 4% w/v Uranyl Acetate (UA) and a UA-Methylcellulose solution for 10 min on ice. The grids were dried at room temperature and viewed using a Tecnai 10 transmission electron microscope (FEI, Eindhoven, Netherlands).

### 2.5. Western Blot Analysis

The exosome-rich pellet and a human hepatocellular carcinoma Huh-7 whole cell extract (control) were resuspended in 1X RIPA buffer, separated on a polyacrylamide gel, and transferred to a polyvinylidene difluoride (PVDF) membrane. The membrane was blocked with bovine serum albumin and incubated first with a CD63 antibody (SBI System Biosciences, USA) or a CD9 antibody (SBI System Biosciences, USA) and then with a goat anti-rabbit HRP secondary antibody (SBI System Biosciences, USA). The proteins were detected by enhanced chemiluminescence (Thermo Scientific, Pierce, USA).

### 2.6. Cell Collection

Huh-7 cells were cultured in DMEM medium containing 10% fetal bovine serum, 100 U/mL penicillin, and 100 g/mL streptomycin at 37°C with 5% CO_2_. When the cells reached approximately 90% confluence (in total, approximately 1.8 × 10^8^ cells), they were collected and washed three times with 1X PBS.

### 2.7. Candidate Reference Genes and Primer Design

Nine miRNAs (*miR-26a*,* miR-221*,* miR-22* ^*^,* miR-181a*,* miR-181c*,* miR-16*,* miR-103*,* miR-191*, and* let-7a*) and two small RNAs (*5SrRNA* and* U6snRNA*) were selected as candidate reference genes to normalize the miRNA RT-qPCR data [10, 15, 16]. The selections were based on the published stability values of these genes. The primer sequences for the candidate reference genes, along with their corresponding accession numbers, are listed in Table S1 (see Supplementary Material available online at http://dx.doi.org/10.1155/2015/893594). Primers for* 5SrRNA* and* U6snRNA* were purchased from RiboBio (Guangzhou, Guangdong, China). The NCBI (http://www.ncbi.nlm.nih.gov/) and miRBase (http://www.mirbase.org/) databases were used to search for available gene sequences, and Primer5 software was used to design the primers. The reaction conditions were optimized by determining the optimal annealing temperature and primer concentration.

### 2.8. RNA Extraction and Reverse Transcription

Exosome-rich pellets were resuspended in 200 *μ*L of 1X PBS and lysed with 1 mL of QIAzol (Qiagen GmbH, Hilden, Germany). RNA was isolated using a miRNeasy Serum/Plasma Kit according to the manufacturer's protocol for liquid samples (Qiagen GmbH, Hilden, Germany). Each RNA sample was eluted in the same volume (volume normalization) after extraction from a given volume of serum (250 *μ*L) and reverse transcribed to cDNA using the GoScript Reverse Transcription System (Promega, USA).

### 2.9. Quantitative Real-Time PCR

Quantitative PCR was performed in 96-well reaction plates with a StepOne Plus Real-Time PCR System (Applied Biosystems, Foster City, USA). The final reaction volume was 20 *µ*L, which included 10 *µ*L of SYBR Select Master Mix (Applied Biosystems, Foster City, USA), 250 nM of each primer, and 2 *µ*L of a 1 : 5 dilution of cDNA. The following thermal cycling conditions were utilized: 1 cycle at 50°C for 2 min, 1 cycle at 95°C for 2 min, and 40 cycles of amplification at 95°C for 15 s and 60°C for 1 min. The threshold cycle (Ct) was calculated using Applied Biosystems SDS software 2.1 (threshold value, 0.38).

### 2.10. PCR Efficiency

*(E) *Standard curves were generated to calculate the RT-qPCR efficiency using 10-fold serial dilutions from a cDNA pool [17]. Duplicate standard curves were included in all the qPCR assays. The obtained individual Ct values were plotted against the logarithm of the dilution factor, and both Pearson's correlation coefficient (*R*) and PCR efficiency (*E*) for each assay were determined from the respective plots. The *R* ^2^ and *E* values were calculated using GenEx Standard software (BioMCC, Freising, Germany). The amplification efficiency was calculated using the following formula: efficiency (%) = (*E* − 1) × 100. In this study, the Minimum Information for Publication of Quantitative Real-Time PCR Experiments (MIQE) guidelines [17] were followed; these guidelines promote experimental consistency and transparency and increase the reliability of the obtained results (Table S2).

### 2.11. Statistical Analysis

The geNorm and NormFinder algorithms were used to calculate the expression stabilities of the candidate reference genes. The geNorm algorithm ranks the tested genes based on their stability measure (*M*). Genes with the lowest *M* are the most stably expressed, whereas the highest *M* values indicate the least stable expression. An *M* value of ≤1.5 indicates a stably expressed gene. To determine the optimal number of control genes for normalization, the pairwise variation, *Vn*/*n* + 1, was calculated as the geometric mean of the relative expression values of the reference genes in the different samples based on the normalization factors (NF_n_ and NF_*n*+1_) [11]. Although the program recommended *Vn*/*n* + 1 values of less than 0.15 for proper normalization, this requirement is not absolute. Other studies have reported no significant improvement with *Vn*/*n* + 1 values exceeding 0.2 [18]. NormFinder is based on an ANOVA mathematical model and calculates the stabilities of candidate reference genes based on the intra- and intergroup variations. A lower stability value indicates a more stably expressed gene [19]. In this study, the NormFinder results in GenEx Standard (BioMCC, Freising, Germany) indicated the optimal number of reference genes by calculating the Accumulated Standard Deviation (Acc.S.D.) based on A. The lowest values correspond to the optimum number of housekeeping genes in the normalization factor to produce accurate and reliable normalization, which permits their ranking according to expression stability. In addition, because the amplification efficiency of each gene is different, the Ct values were used as input data after correcting for their respective amplification efficiencies using GenEx

$$\begin{array}{cc} \left(\text{A}\right) & \text{Acc}.\text{S}.\text{D}.=\frac{1}{n}\sqrt{\overset{n}{\underset{i=1}{\sum }}{\text{SD}}_{i}^{2}}. \end{array}$$

Here, the Acc.S.D. is based on *n* reference genes and is calculated as the geometric average of the *n* raw reference gene quantities for any given gene *i*.

GenEx Standard software was used to calculate the expression of target miRNAs relative to suitable normalization genes. All the data were corrected based on their respective amplification efficiencies, and the relative quantification (RQ) values were calculated for all the samples within each group. Next, the RQ values in the each group were adjusted to the maximum value and log_2_-transformed for analysis. The distribution of continuous data was determined using the Kolmogorov-Smirnov test, and *t-*tests were used to identify significant differences in target miRNA expression between two groups. If these data were not normally distributed, we used nonparametric hypothesis tests to determine their distribution. The statistical tests were two-sided, and *P* values of less than 0.05 were considered statistically significant. The data were analyzed using SPSS 19.0 (IBM, USA).

## 3. Results

### 3.1. Characterization of Serum-Derived Exosomes

**Figure 1:**
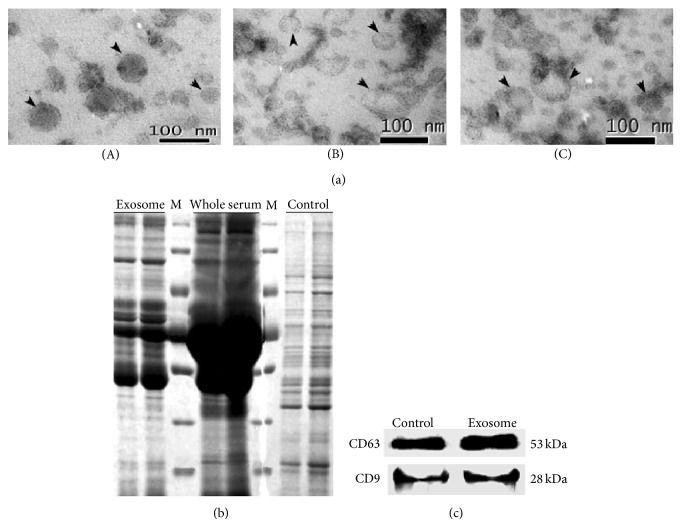
Identification of exosomes circulating in serum. (a) Exosome size was evaluated by Transmission Electron Microscopy (TEM). The sample sources were healthy controls (A), CHB patients (B), and HCC patients (C). (b) Coomassie staining of serum-derived exosomal proteins, whole serum, and Huh-7 whole cell protein extracts (control). “M” represents a specific molecular marker ladder (Fermentas) from 130 kDa to 26 kDa. (c) Huh-7 whole cell extracts and exosomes were lysed with 1X RIPA buffer. The exosomal marker tetraspanin proteins CD63 and CD9 were analyzed by Western blot.

The successful isolation of exosomes from serum is necessary to accurately quantify exosomal miRNA. In our study, we obtained serum exosomes using ExoQuick exosome precipitation solution. Under an electron microscope, exosomes are small membrane-bound vesicles with diameters ranging from 40 to 100 nm (Figure 1). The exosome surface contains the tetraspanin family proteins CD63 and CD9, thereby supporting the endosomal origin of exosomes [4, 20]. Therefore, we used these markers to identify pellets containing exosomes. Accordingly, our Western blotting analysis demonstrated that all pellets contained CD63 and CD9 (Figure 1), clearly indicating that these pellets included exosomes.

### 3.2. Selection of Candidate Reference Genes and Specific Amplification

The optimal reference gene(s) were identified from among nine stable miRNAs (*miR-16*,* miR-103*,* miR-22* ^*^,* miR-26a*,* miR-221*,* miR-181a*,* miR-181c*,* let-7a*, and* miR-191*) and two small RNAs (*5SrRNA* and* U6snRNA*) [10, 15, 16] that were selected based on the literature. None of these candidates reside within the same gene cluster, which reduces the likelihood of including coregulated miRNAs in the analysis [21].

We subsequently determined the reliability of quantifying these candidate genes. The gene-specific PCR amplification efficiency (*E*%) was calculated from the slope of the standard curve. The *E* values for the 11 reference genes varied from 91.9% to 98.2%, and the regression correlation coefficients (*R* ^2^) ranged from 0.988 to 0.999 (Table S2). The melting curves exhibited single peaks corresponding to unique amplicons.

### 3.3. Expression of Candidate Reference Genes

**Figure 2:**
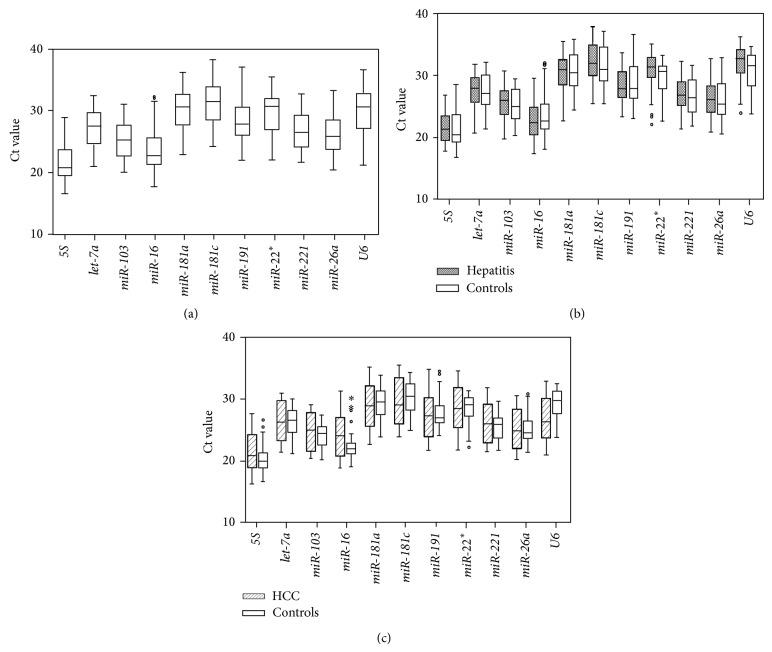
Expression of candidate reference genes in circulating exosomes. RT-qRCR analyses were performed on serum exosomal miRNAs. The box plot graphs of the Ct values for each reference gene illustrate the interquartile range (box) and median. The whisker plot depicts the range of the values. Circles indicate outliers. (a) All the studied samples. (b) Hepatitis B patients and age- and gender-matched healthy volunteers. (c) HCC patients and age- and gender-matched control individuals.

We measured the expression of the 11 reference candidates by RT-qPCR in serum exosome samples from three groups of subjects: healthy persons, CHB patients, and HCC patients. The Ct value ranges are presented in Figure 2 for each transcript amplified from each biological replicate.* miR-181c* had the lowest expression, whereas* 5S* had the highest expression among all the samples (Figure 2).

### 3.4. Analysis of the Stabilities of Serum Exosomal Reference Genes

**Figure 3:**
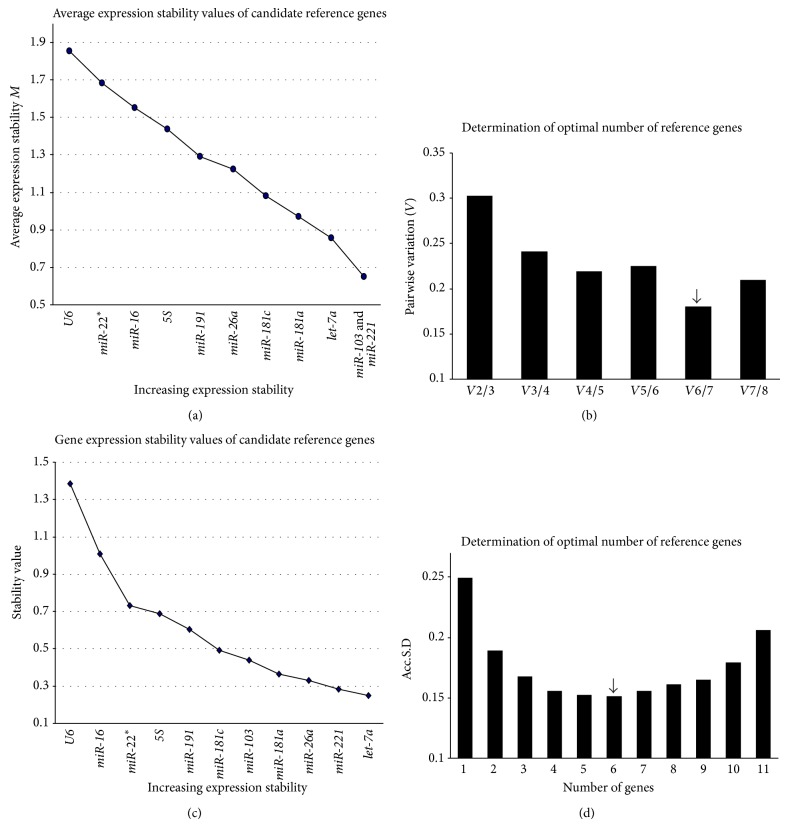
The stability of the candidate genes and the optimal number of reference genes for transcript normalization in all the samples. (a) Expression stabilities of the reference genes from the least stable (*left*) to the most stable (*right*) as analyzed by geNorm. (b) After excluding* U6*,* miR-22* ^*^, and* miR-16* due to *M* > 1.5, the pairwise variations (*Vn*/*n* + 1) were analyzed for all three experimental groups.* miR-221*,* miR-103*,* let-7a*,* miR-181c*,* miR-181a*, and* miR-26a* (RG-6) were recommended as the optimal combination of reference genes. (c) The expression stability values were calculated using NormFinder. A lower stability value indicates more stable expression. (d) The gene expression Acc.S.D. was analyzed using NormFinder. The lowest Acc.S.D. value indicated that the optimal number of reference genes was 6 (RG-6).

The stability of the eleven candidate reference genes was evaluated using the geNorm [11] and NormFinder [19] algorithms according to their respective manuals. The *M* values of the examined reference genes in the three groups of serum exosome samples are presented in Figure 3(a). The *M* values were less than 1.5 for all candidate reference genes, with the exception of* miR-16*,* miR-22* ^*^, and* U6*, suggesting that they were not reliable reference genes. Excluding the unstable genes, the *Vn*/*n* + 1 analysis indicated that the lowest *V* value was *V*6/7 (0.18). Therefore, geNorm recommended six miRNAs (*miR-221*,* miR-103*,* let-7a*,* miR-181c*,* miR-181a*, and* miR-26a*) (RG-6) as the optimal combination of reference genes (Figure 3(b)). NormFinder ranked* let-7a* as the most stably expressed gene, followed by* miR-221*,* miR-26a*,* miR-181a*,* miR-103*, and* miR-181c* (Figure 3(c)). The lowest Acc.S.D. value indicated that the optimal number of control genes was six (RG-6). Notably, the same set of reference genes was recommended by both NormFinder and geNorm (Figure 3(d)).

**Figure 4:**
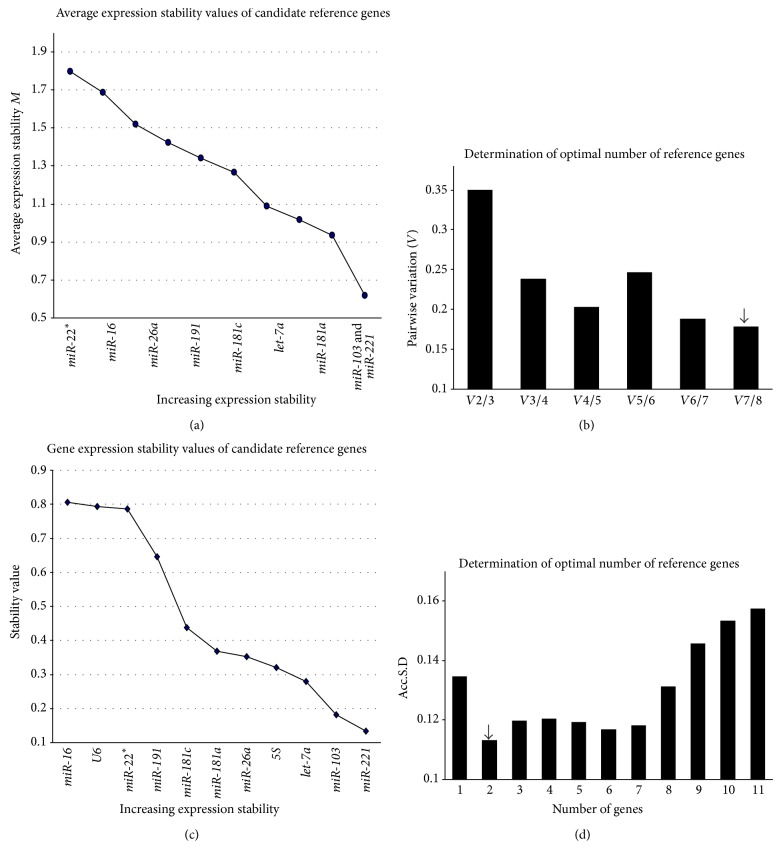
The stability of the candidate genes and the optimal number of reference genes for transcript normalization in subset 1. (a) The stabilities of the reference genes as determined by geNorm. (b) After excluding* U6*,* miR-22* ^*^, and* miR-16* due to *M* > 1.5, we analyzed the pairwise variations (*Vn*/*n* + 1) using geNorm. The combination of* miR-221*,* miR-103*,* let-7a*,* miR-181c*,* miR-181a*,* miR-191*, and* miR-26a* (RG-7) was recommended. (c) The expression stability values were evaluated using NormFinder. (d) In NormFinder, the gene expression Acc.S.D. was analyzed. The lowest Acc.S.D. value indicated that the optimal reference gene set included* miR-221* and* miR-103* (RG-2).

Considering the heterogeneity of liver diseases, we then analyzed each experimental condition individually to identify reference genes that were specific to each disease. We divided the entire dataset into three subsets for the reanalysis. The first subset included CHB patients and healthy controls. The* miR-221*/*miR-103* pair (RG-2) was selected by geNormas as the least variable among all the reference genes (Figure 4(a)). This pair was also selected by NormFinder as the most stable reference gene set for normalizing exosomal miRNA in the two biological groups (Figures 4(c) and 4(d)). As Figure 4(a) shows,* miR-16*,* miR-22* ^*^, and* U6* were highly unstable in the first subset: their average *M* values exceeded the acceptable value of 1.5 (Figure 4(c)). Excluding these unstable genes, geNorm recommended a combination of seven reference genes (*miR-221*,* miR-103*,* let-7a*,* miR-181c*,* miR-181a*,* miR-191*, and* miR-26a*) (RG-7) (Figure 4(b)).

**Figure 5:**
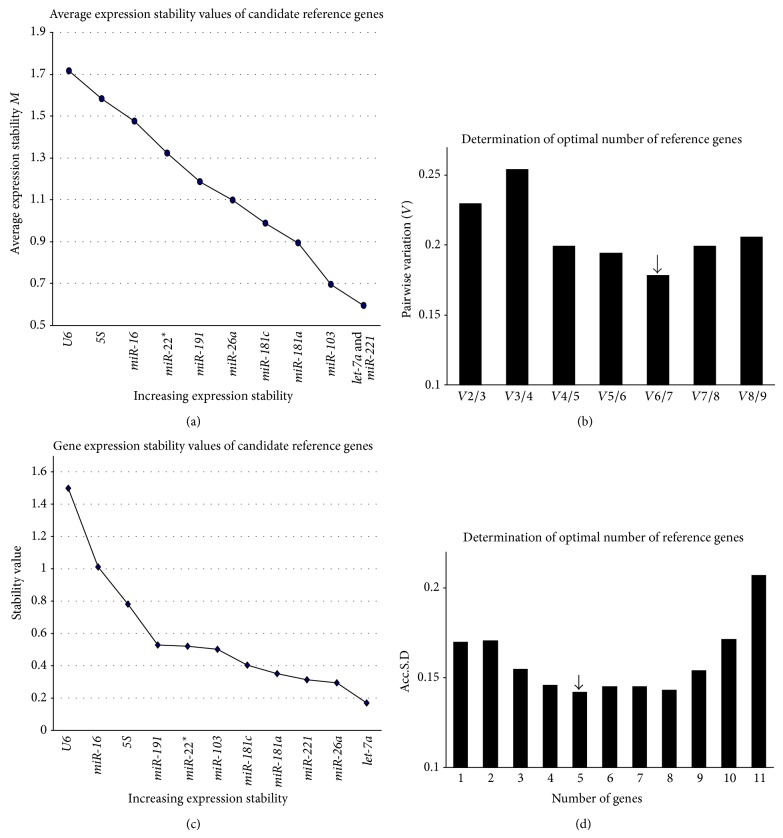
The stability of the candidate genes and the optimal number of reference genes for transcript normalization in subset 2. (a) The expression stability of each gene as analyzed by geNorm. (b) After excluding* U6* and* 5S* due to *M* > 1.5, the pairwise variations (*Vn*/*n* + 1) were analyzed for subset 2. The same six miRNAs (RG-6) were recommended as the optimal combination of reference genes in subset 2. (c) The expression stability values were evaluated using NormFinder. (d) The Acc.S.D. values were analyzed using NormFinder. The lowest value indicated that the optimal number of reference genes was 5 (RG-5).

HCC patients and healthy persons were grouped in the second subset. The most stable genes based on the geNorm analysis were* let-7a* and* miR-221*, and* let-7a* was also considered to be the least variable gene by NormFinder (Figures 5(a) and 5(c)).* 5S* and* U6* were excluded from the set of reference genes suggested by geNorm; the most stable combination based on the geNorm analysis included* miR-221*,* miR-103*,* let-7a*,* miR-181c*,* miR-181a*, and* miR-26a* (RG-6). This combination was also the most stably expressed in all the samples. In contrast to geNorm, NormFinder considered* miR-103* to be less stable and recommended the other five genes (RG-5) as the optimal reference gene set.

**Figure 6:**
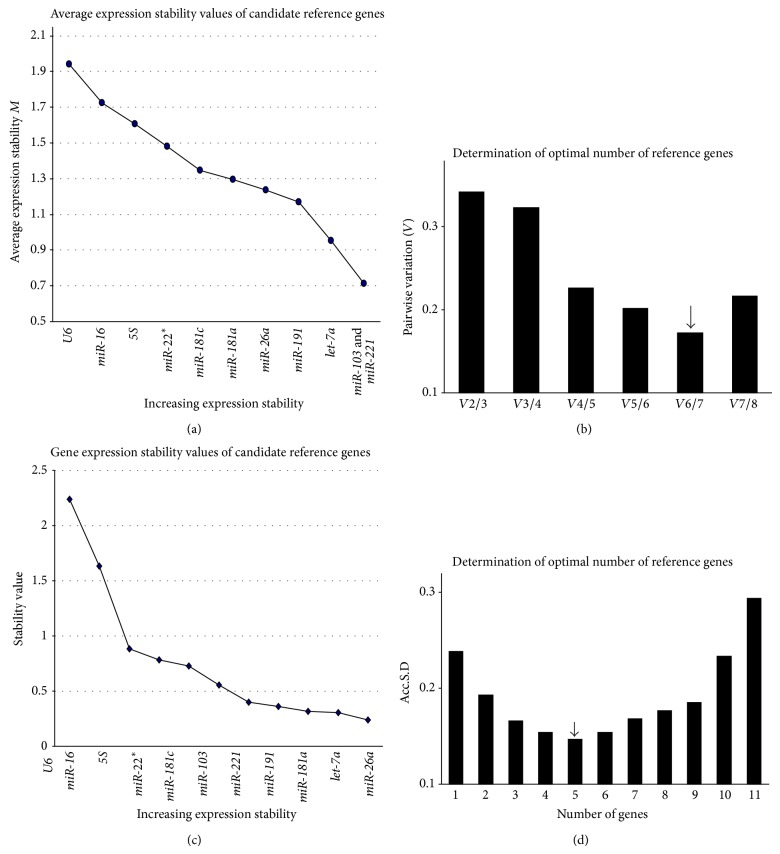
The stability of the candidate genes and the optimal number of reference genes for transcript normalization in subset 3. (a) The expression stability of each gene as analyzed by geNorm. (b) After excluding* U6*,* miR-16*, and* 5S* due to *M* > 1.5, the pairwise variations (*Vn*/*n* + 1) were analyzed for subset 3. The* miR-221*,* miR-103*,* let-7a*,* miR-181a*,* miR-191*, and* miR-26a* (RG-6d) were recommended. (c) The expression stability values were evaluated using NormFinder. (d) The Acc.S.D. values were analyzed using NormFinder. The lowest value indicated that the optimal number of reference genes was 5 (RG-5d).

The third subset was combined by HCC and CHB patients. geNorm excluded three unstable genes and recommended* miR-221*,* miR-103*,* let-7a*,* miR-191*,* miR-26a*, and* miR-181a* (RG-6d) as the least variable set of reference genes (Figures 6(a) and 6(b)). Similar to subset 2, NormFinder found larger intergroup variation of* miR-103* and considered other five genes (RG-5d) as the optimal reference gene set (Figures 6(c) and 6(d)). Briefly, RG-6 and RG-5 differed from RG-6d and RG-5d. The former sets included* miR-181c* but not* miR-191* that presented in the latter sets.

### 3.5. Influence of Reference Gene Selection on the Relative Quantification Accuracy

To evaluate the normalization efficiency of the reference genes, we applied different normalization strategies to perform a relative quantification of* miR-21*. Plasma* miR-21* levels correlated significantly with* miR-21* expression levels in tumor tissues [22]. And previous reports had demonstrated that serum* miR-21* levels of patients with CHB patients were higher than those of patients with HCC and of healthy controls. Thus* miR-21* has strong potential to serve as a novel biomarker for liver injury [23].

**Figure 7:**
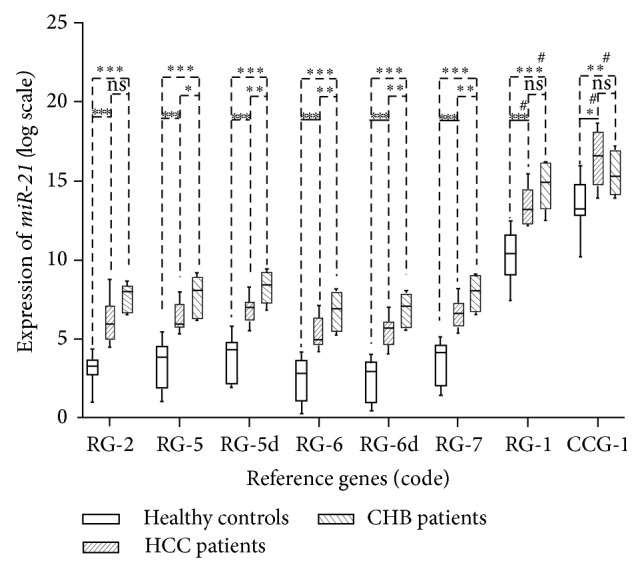
Effects of different reference genes on the normalization of* miR-21* expression. The expression of* miR-21* was measured in serum exosomes from 18 CHB patients, 18 HCC patients, and 18 healthy subjects by RT-qPCR. The difference in* miR-21* expression between groups was analyzed by *t*-test. # represents the probability distribution for two groups of log_2_-converted data that did not obey normal distribution; a nonparametric hypothesis test was used to identify their distribution. Different levels of statistical significance are denoted with asterisks (∗). “ns” represents the statistics results indicating that the two groups of log_2_-converted data were not significantly different.

Different normalization approaches were used to assess* miR-21* expression in the three groups. The expression of exosomal* miR-21* transcript was significantly increased in CHB patients compared with the other two groups, when the reference gene combinations recommended by the two algorithms were utilized. As a comparison, when* miR-181c* (RG-1) or* U6* (CCG-1) was used as reference gene, there was no significant difference in* miR-21* expression between patients with HCC and CHB (Figure 7).

## 4. Discussion

The interest in using miRNAs within circulating exosomes as noninvasive biomarkers has increased rapidly. The selection of suitable reference genes as normalization factors is necessary to accurately compare exosomal miRNA transcripts. Two types of reference genes have recently been used to normalize miRNA expression data: synthetic miRNA molecules (spike-in controls) and endogenous control genes. External spike-in controls are not assumption-free; it is assumed that the experimenter starts with the same quantity of equal quality template. Normalization factors that are based on endogenous miRNA are therefore preferred.

**Figure 8:**
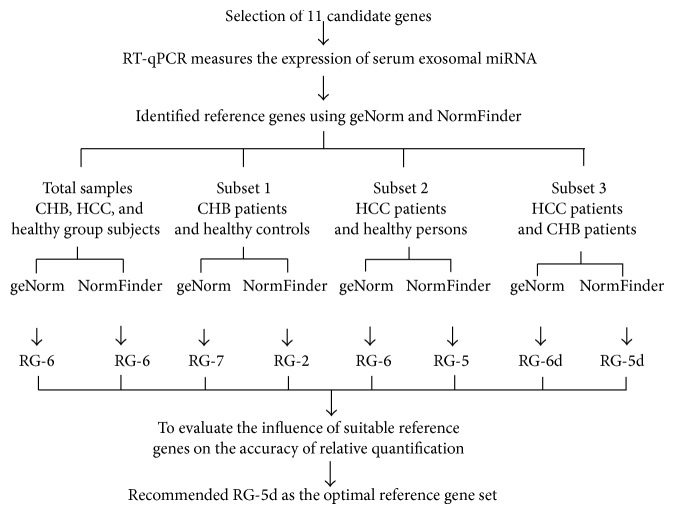
Flowchart illustrating the approach. This figure shows the general strategy utilized to identify a pool of reference gene candidates for different groups of subjects and to determine reference gene sets using geNorm and NormFinder for real-time RT-qPCR experiments.

In this study, we selected optimal reference genes in four steps to improve the accuracy with which differences in serum exosomal miRNAs between patients with liver disease and healthy controls can be ascertained (Figure 8). To the best of our knowledge, the present study is the first systematic investigation of suitable reference genes for RT-qPCR data analysis of circulating exosomal miRNA in liver disease.

Currently, the majority of HCC is thought to be a consequence of chronic liver inflammation. Viral and host inflammation-related factors are important predictors of HCC prognosis after surgery [24]. We measured 11 reference genes in serum exosomes from CHB or HCC patients and healthy controls. The geNorm and NormFinder algorithms demonstrated that* miR-221*,* miR-103*,* let-7a*,* miR-181c*,* miR-181a*, and* miR-26*a (RG-6) were the most stably expressed reference genes in all the samples. Among the top reference genes,* miR-221* was identified as the most stable, followed by* miR-103* and* let-7a*. Similar results were observed by Qi et al., who found that* miR-221* was not differentially expressed in the sera of HCC patients and healthy subjects [25]. Additionally, plasma* miR-103* ranked as the most stably expressed reference gene for acetaminophen hepatotoxicity in a study by Wang et al. [26]. Furthermore, our results corroborate a recently published paper by Arroyo et al., in which they demonstrated that* let-7a* was predominantly exosome-associated and might be of cellular origin [27]. In our study,* let-7a* was ranked as the third most stable reference gene. Similarly,* let-7a* ranked among the top most stable reference genes in a breast cancer study [28]. Timoneda and colleagues demonstrated that* miR-26a* was the most stable miRNA in porcine liver and that the combination of* miR-26a*,* let-7a*, and* miR-103* was recommended as the optimal reference gene set for liver studies [29].

Our study confirmed the importance of reference gene optimization for each RT-qPCR experiment. Different experimental settings can result in changes in the stability of the reference genes. In our study, all the candidate reference genes were analyzed in three subsets using geNorm and NormFinder. We identified five different reference gene combinations. There are two brief reasons for this distinction. First, the NormFinder program estimates both the intra- and intergroup variation and combines them into the stability value. However, geNorm calculates an *M* value predominantly based on the intragroup variation, so it must exclude coregulated or coexpressed genes. This discrepancy has been previously described [11, 19, 30]. Second, and more importantly, different results are obtained when optimizing reference genes from different diseases by RT-qPCR. As previous studies have demonstrated, there is no “one” reference gene that can be used across all experiments [11, 12, 28].

The practicality of a normalization gene also influences the selection of an optimal reference gene. Therefore, we tested the suitability of the different approaches with* miR-21*. Except for RG-2, other combinations of reference genes recommended by NormFinder or geNorm were propitious to assess the biological variability of target miRNAs (Figure 7). It was worth noting that RG-2 was found out in subset 2 which included CHB patients and healthy controls. There was great possibility that RG-2 could not normalize miRNA in group of patients with CHB and HCC. In addition, we chose* miR-181c* (RG-1) as a control gene. Although there have been many reports on screening miRNA expression in serum samples or tissue samples from patients with liver disease by PCR array analysis, none of these studies identified a significant change in* miR-181c* in hepatopathy samples. In our study,* miR-181c* was considered to be a stable reference gene. However, we found that* miR-181c* introduced bias into the analysis and led to the misinterpretation of* miR-21* expression in HCC patients compared with CHB patients.

We also selected another common housekeeper gene like* U6* (CCG-1) for the relative quantification of target miRNAs. Our results indicated that the normalization of exosomal miRNA expression using CCG-1 was inappropriate. Compared with the results of Wang et al.'s study [8], we did not verify a higher* miR-21* expression in HCC than in CHB when using the same reference gene* U6* (CCG-1). This discrepancy may be due to subject selection. We demonstrated wide differences in the expression of exosomal* U6* in our study. This unstable expression led to differential results when* U6* was used as a normalization factor for* miR-21*.

In summary, we presented the first experimentally validated optimal reference genes to normalize miRNA expression in serum-derived exosomes. All of the combinations proposed in this study were appropriate for normalization. However, RG-2was not reliable as a reference gene across the groups, especially when used to normalize target genes with smaller fold changes. RG-5 had lower sensitivity for the comparability of* miR-21* expression between CHB patients and HCC patients. Therefore, for comprehensive investigation into the progression of CHB to HCC, we considered that the combination of* miR-221*,* let-7a*,* miR-191*,* miR-26a*, and* miR-181a* (RG-5d) was the optimal reference gene set, on account of the technical and economic advantages of using a smaller number of reference genes. Furthermore, it was inappropriate to normalize the data with* U6* or* miR-181c*. The present study, which identified optimal reference genes, will improve studies that monitor the progression of hepatitis and will help identify noninvasive biomarkers to diagnose early stage hepatic carcinoma.

## Supplementary Material

Supplemental Table 1 shows The primer sequences for the candidate reference genes, along with their corresponding accession numbers and each of Real-Time PCR Experiments amplification efficiency.
Supplemental Table 2 shows our RT-qPCR analyses followed MIQE guidelines. These guidelines promote experimental consistency and transparency and increase the reliability of the obtained results.

## Figures and Tables

**Table 1:** Clinical characteristics of subjects enrolled in the study.

	Healthy controls (*n* = 50)		Hepatitis B patients (*n* = 50)		HCC patients (*n* = 50)	
	Number	*n* (%)	Number	*n* (%)	Number	*n* (%)
Gender						
Male	39	78	37	74	41	82
Female	11	12	13	26	9	18
HBV status						
HBsAg+	0	0	50	100	38	76
HBsAg−	50	100	0	0	12	24
Age (median, yr)						
<40	6	12	5	10	4	8
40–60	36	72	35	70	36	72
>60	8	16	10	20	10	20
