# Supplementary material for: Identification of Endogenous Controls for Analyzing Serum Exosomal miRNA in Patients with Hepatitis B or Hepatocellular Carcinoma
Source: Dis Markers. 2015 Feb 26;2015:893594. doi: 10.1155/2015/893594 (PMC4357047; doi:10.1155/2015/893594)
Supplement: Supplementary file 1 — Supplemental Table 1 shows The primer sequences for the candidate reference genes, along with their corresponding accession numbers and each of Real-Time PCR Experiments amplification efficiency. Supplemental Table 2 shows our RT-qPCR analyses followed MIQE guidelines. These guidelines promote experimental consistency and transparency and increase the reliability of the obtained results. [file 893594.f1.pdf]

TABLE S1: Descriptions of gene-specific RT-qPCR assays.

Gene name	Accession number		Primer Sequences	E(%)	R^2
Candidate reference miRNAs					
miR-16	MIMAT0000069	RT	5'GTCGTATCCAGTGCAGGGTCCGAGGTATTCGCACTGGATACGACCGCCAA 3'	98.2	0.998
		F	5' GCCCGTAGCAGCACGTAAATATT 3'		
miR-103	MIMAT0000101	RT	5'GTCGTATCCAGTGCAGGGTCCGAGGTATTCGCACTGGATACGACTCATAG 3'	96.3	0.999
		F	5' GGTATAGCAGCATTGTACAGGGC 3'		
miR-191	MIMAT0000440	RT	5'GTCGTATCCAGTGCAGGGTCCGAGGTATTCGCACTGGATACGACCAGCTG 3'	96.8	0.999
		F	5' GGTAACAACGGAATCCCAAAAGC 3'		
let-7a	MIMAT0000062	RT	5'GTCGTATCCAGTGCAGGGTCCGAGGTATTCGCACTGGATACGACAACTAT 3'	94.4	0.997
		F	5' GCCGCTGAGGTAGTAGGTTGTAT 3'		
miR-26a	MIMAT0000082	RT	5'GTCGTATCCAGTGCAGGGTCCGAGGTATTCGCACTGGATACGACAGCCTA 3'	91.9	0.993
		F	5' GCCGCTTCAAGTAATCCAGGATA 3'		
miR-221	MIMAT0000278	RT	5'GTCGTATCCAGTGCAGGGTCCGAGGTATTCGCACTGGATACGACGAAACC 3'	97.2	0.996
		F	5' GCCTAAGCTACATTGTCTGCTGG 3'		
miR-22*	MIMAT0004495	RT	5'GTCGTATCCAGTGCAGGGTCCGAGGTATTCGCACTGGATACGACTAAAGC 3'	93	0.997
		F	5' GCATCAGTTCTTCAGTGGCAAGC 3'		
miR-181a	MIMAT0000256	RT	5'GTCGTATCCAGTGCAGGGTCCGAGGTATTCGCACTGGATACGACACTCAC 3'	95.8	0.998
		F	5' CGTGCTAACATTCAACGCTGTC 3'		
miR-181c	MIMAT0000258	RT	5'GTCGTATCCAGTGCAGGGTCCGAGGTATTCGCACTGGATACGACACTCAC 3'	93.8	0.997
		F	5' GCATAACATTCAACCTGTCGGT 3'		
**Interest miRNA**					
miR-21	MIMAT0000076	RT	5'GTCGTATCCAGTGCAGGGTCCGAGGTATTCGCACTGGATACGACTCAACA 3'	98	0.996
		F	5' GCCGTTAGCTTATCAGACTGATG 3'		
	miRNA universal R		5' GTGCAGGGTCCGAGGT 3'		

TABLE S2: Amplicon parameters derived from the RT-qPCR data analysis.

Gene	Y-Intercept	Slope	PCR efficiency (%)	SE[#](Efficiency)	R^2	Linear dynamic range[$]	Ct variation at the lowest limit(SD)[&]	Ct of NTC[※]
miR-16	12.29	-3.366	98.2	0.012	0.998	12.04-28.97	0.18	UD
miR-22*	16.4	-3.501	93	0.018	0.997	16.40-38.43	0.78	UD
U6	15.71	-3.534	91.9	0.037	0.988	15.97-38.00	0.09	UD
5S	12.32	-3.475	94	0.035	0.99	11.51-33.27	0.58	38.4476
miR-181c	16.22	-3.48	93.8	0.02	0.997	16.12-37.99	0.9	38.7177
miR-181a	16.05	-3.426	95.8	0.016	0.998	16.16-37.31	0.6	UD
miR-221	15.76	-3.39	97.2	0.024	0.996	15.75-32.38	0.1	UD
miR-26a	16.52	-3.532	91.9	0.03	0.993	16.00-34.32	0.59	UD
let-7a	16.64	-3.463	94.4	0.018	0.997	16.55-34.21	0.18	UD
miR-191	15.97	-3.401	96.8	0.011	0.999	15.94-33.02	0.19	39.5648
miR-103	12.72	-3.414	96.3	0.012	0.999	12.82-30.21	0.12	UD
miR-21	13.45	-3.48	93.8	0.01	0.996	13.43-32.02	0.25	UD

[#] The SE value is the mean squared error of the single data points fit to the regression line and is calculated by the GenEx Standard software.

[$] The linear dynamic range represents the range of the Ct values between the highest and the lowest concentration of the linear interval of the calibration curve.

[&]Ct variations given as the SD at the endpoint of the linear dynamic range that correspond to the lowest concentration in the linear interval of the calibration curve.

[※]UD represents undetermined data.
